# The Therapeutic Role of ADSC-EVs in Skin Regeneration

**DOI:** 10.3389/fmed.2022.858824

**Published:** 2022-06-09

**Authors:** Yixi Wang, Lihui Cheng, Hanxing Zhao, Zhengyong Li, Junjie Chen, Ying Cen, Zhenyu Zhang

**Affiliations:** ^1^Department of Plastic and Burn Surgery, West China Hospital, Sichuan University, Chengdu, China; ^2^Department of Central Sterile Supply, West China Hospital, Sichuan University, Chengdu, China

**Keywords:** skin regeneration, extracellular vesicles, adipose-derived stem cells, stem cells therapy, wound healing

## Abstract

Large skin defects caused by burns, unhealing chronic wounds, and trauma, are still an intractable problem for clinicians and researchers. Ideal skin regeneration includes several intricate and dynamic stages of wound repair and regeneration of skin physiological function. Adipose-derived stem cells (ADSCs), a type of mesenchymal stem cells (MSCs) with abundant resources and micro-invasive extraction protocols, have been reported to participate in each stage of promoting skin regeneration via paracrine effects. As essential products secreted by ADSCs, extracellular vesicles (EVs) derived from ADSCs (ADSC-EVs) inherit such therapeutic potential. However, ADSC-EVs showed much more clinical superiorities than parental cells. ADSC-EVs carry various mRNAs, non-coding RNAs, proteins, and lipids to regulate the activities of recipient cells and eventually accelerate skin regeneration. The beneficial role of ADSCs in wound repair has been widely accepted, while a deep comprehension of the mechanisms of ADSC-EVs in skin regeneration remains unclear. In this review, we provided a basic profile of ADSC-EVs. Moreover, we summarized the latest mechanisms of ADSC-EVs on skin regeneration from the aspects of inflammation, angiogenesis, cell proliferation, extracellular matrix (ECM) remodeling, autophagy, and oxidative stress. Hair follicle regeneration and skin barrier repair stimulated by ADSC-EVs were also reviewed. The challenges and prospects of ADSC-EVs-based therapies were discussed at the end of this review.

## Introduction

The skin, the largest organ of the human body, protects the body from exogenous irritation and pathogen invasion as the first barrier between organisms and the environment. Skin damage caused by diseases or trauma threatens the defensive function, leading to the suffering of patients and the burden of public health care (1, 2). Repair of skin, including both structural integrity and physiological function, is essential to maintain its protective property, which remains intractable for clinicians and researchers. Stem cells have been reported to possess considerable potential for skin regeneration through multiple mechanisms (3–5). Adipose-derived stem cells (ADSCs) are a promising type of mesenchymal stem cells (MSCs) for skin regeneration, with abundant resources among human tissue and minimally invasive extraction protocols. However, some severe complications impact the application of stem cells since they are large and sticky, such as elevation in pulmonary arterial pressure or even vascular embolism, along with potential oncogenesis and ethical issues (6, 7). Moreover, some properties of stem cells might impair the beneficial effect of stem cells. For example, restricted delivery of stem cells and uncertain differentiation to proinflammatory or anti-inflammatory phenotype of stem cells in inflammatory conditions might lead to no benefit or even negative effect in the treatment of acute kidney injury after cardiac surgery (8).

**Figure 1:**
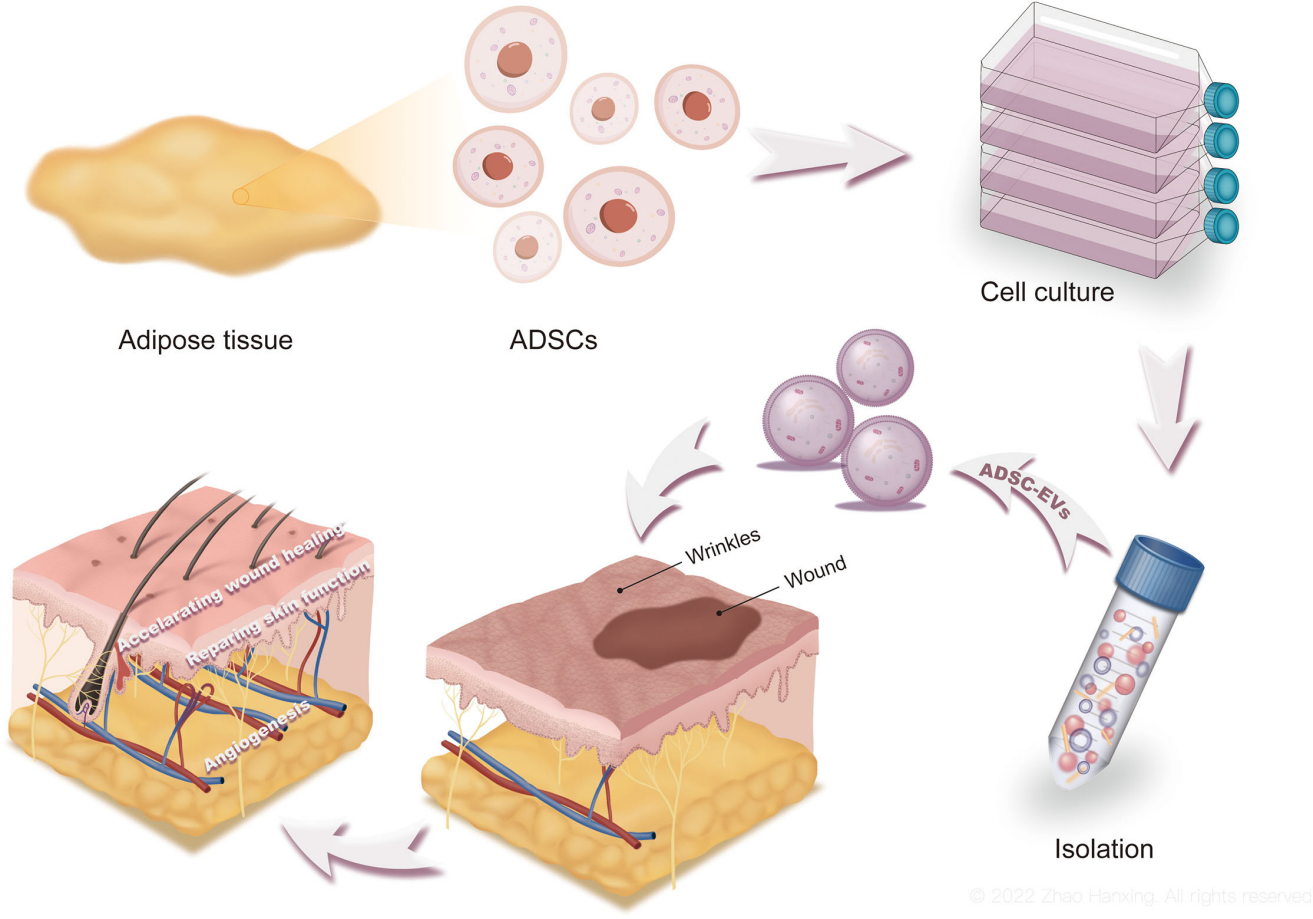
The production, functions, and applications of ADSC-EVs. Culture medium of ADSCs was collected and processed to obtain ADSC-EVs. By accelerating wound healing and repairing skin function, ADSC-EVs promote skin regeneration. ADSC-EVs are promising for clinical applications as well.

Extracellular vesicles (EVs) are natural particles with a phospholipid bilayer membrane secreted by almost all types of cells during vital activities (9). Transferring proteins, nucleic acids, and lipids to recipient cells, EVs derived from stem cells have been deemed to be intercellular communicators and functional executors (10, 11). Compared to stem cells, EVs possess more advantages for clinical application. EVs are safer owing to their smaller size and non-tumorigenic. Because EVs show no immunogenicity and can be stored at −80°C, they are available once patients need, avoiding waiting for cell culture as autologous stem cells therapy (12). In recent years, considerable research efforts on EVs derived from ADSCs (ADSC-EVs) have indicated that ADSC-EVs have a positive impact on skin regeneration, similar to their parental cells. Moreover, ADSC-EVs manifested a superior impact on wound healing than EVs derived from other stem cells, which might be due to their robust angiogenic effect (13). ADSC-EVs might accelerate skin wound repair by participating in inflammation, angiogenesis, cell proliferation, and extracellular matrix (ECM) remodeling (14, 15), regulating cell apoptosis and autophagy (16, 17), and relieving oxidative stress in the wound microenvironment (18). The regeneration of skin appendages and recovery of physiological functions are also promoted by ADSC-EVs (19, 20), which is essential for ideal skin regeneration. Current documents have summarized the promoting effects of ADSC-EVs in skin regeneration, mostly focusing on mechanisms in wound healing but hardly with functional repair involved. In this review, the profile of ADSC-EVs, mechanisms in the promotion of skin regeneration, and potential for clinical applications are discussed (Figure 1). The existing challenges and prospects of ADSC-EVs in regenerative medicine are also discussed here. We hope this work replenishes current comprehension of how ADSC-EVs generate and work, and provides potential inspiration for future research on regenerative medicine.

## Extracellular Vesicle From Adipose-Derived Stem Cells

ADSCs are a subtype of MSCs isolated from adipose tissues with self-renewal and multiple differentiation properties. Through paracrine of a variety of cytokines, ADSCs are known as powerful therapeutics utilized in regenerative medicine (21, 22). In addition to direct secretion, ADSC-EVs have been reported to be functional executors of ADSCs (23). As products of ADSCs during biological activities, ADSC-EVs encapsulate cargoes, including DNA, RNA, proteins, and lipids produced by ADSCs, acting as intercellular communicators and biomolecule transporters (24).

### Classification and Biogenesis of EVs

**Figure 2:**
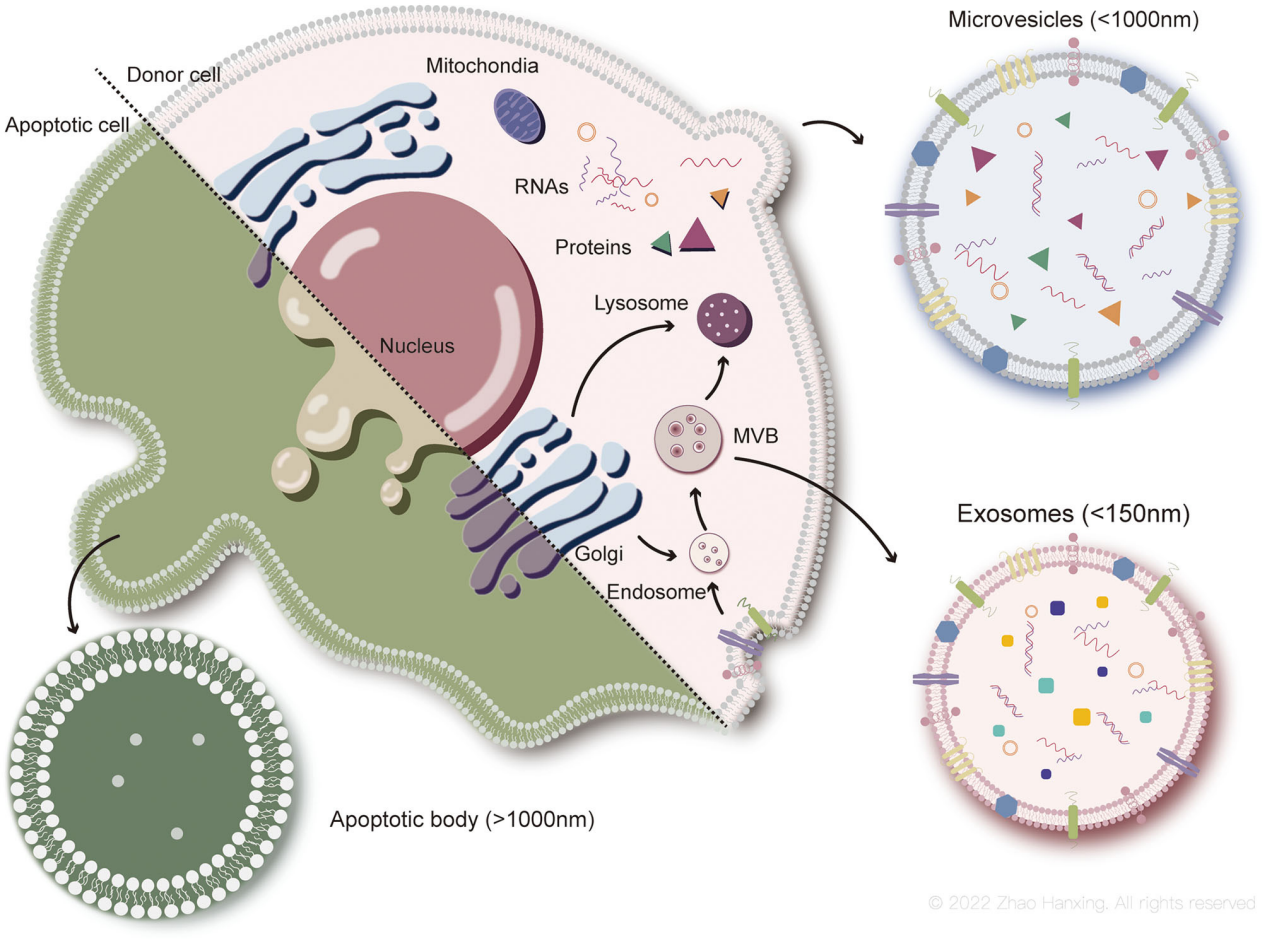
Biogenesis of each subtype of EVs. Exosomes (<150 nm in size) originate from endosome. Microvesicles (up to 1,000 nm) generate from plasma membrane. Apoptotic bodies (more than 1,000 nm) are particles of apoptotic cells disassembly.

EVs are a generic term for particles with lipid bilayer membranes released by cells in natural activities and are divided into three main subtypes based on the current understanding of their biogenesis, size, and content: exosomes (<150 nm in size), microvesicles (up to 1,000 nm), and apoptotic bodies (more than 1,000 nm) (25–27). Exosomes and microvesicles seem to be generated by almost all types of viable cells (28) and are major subtypes of EVs studied in regenerative medicine to date. Apoptotic bodies, the relatively larger group in size, are products of cell apoptosis and encapsulate contents of cells disassembly. The biogenesis of the three main EV-subtypes is shown here (Figure 2).

The biogenesis of exosomes is a complex process. First, endocytosis of the plasma membrane forms the early sorting endosomes, carrying surface proteins and lipids. Early endosomes subsequently transform to late endosomes under interaction with the Golgi complex (28). Exosomes originate from the inward budding of the endosomal membrane as intraluminal vesicles (IVLs) during maturation of multivesicular endosomes (MVBs), which then enter lysosomes for degradation, or fuse with the plasma membrane to be released as exosomes (27). Exosomal membrane components are derived from plasma or the Golgi complex before the formation of IVLs in endosomes. The cargoes sorted into exosomes are heavily dependent on endosomal sorting mechanisms, which involve the endosomal sorting complex required for transport (ESCRT) proteins (29). ESCRT-independent mechanisms have also been demonstrated, including ceramide and its metabolites and tetraspanin family members (30–32).

Microvesicles released by healthy cells originate from the outward budding of the plasma membrane. Phospholipids on the plasma membrane rearrange with activation of scramblase by the inflow of Ca^2+^, in which phosphatidylserine flips from the inner leaflet of the bilayer to the outer leaflet. Consequently, budding of the plasma membrane and degradation of the cytoskeleton occur, forming microvesicles (33). However, biogenesis of microvesicles can proceed without the rearrangement of phospholipids (34), indicating that other mechanisms may also be involved, such as the participation of cholesterol-rich lipid rafts (35).

Although the destinies differ between parental cells of apoptotic bodies and microvesicles, the formation of the two subtypes of EVs involves similar changes in the plasma membrane. Biogenesis of apoptotic bodies is described as three sequential well-coordinated steps with corresponding morphological changes: plasma membrane blebbing, thin membrane protrusion formation, and fragmentation (36). During blebbing, phosphatidylserine flips from the inner layer of the plasma membrane to the outer layer, which is induced by caspase-activated scramblase (37). Compared to the former two subtypes, cargoes of apoptotic bodies tend to include intact organelles, chromatin, and higher levels of histones (38). However, recent studies have also shown that some organelles might be enclosed by microvesicles (39). Information on cargoes in EVs needs to be enriched with further research.

Biomarkers of EVs include molecules involved in their biogenesis, such as transmembrane proteins anchored to the plasma membrane or endosomal membrane and cytosolic proteins (27). Non-EV proteins co-isolated with EVs are detected to assess the purity of EVs, such as apolipoproteins A1/2 and B, and albumin (26). According to the biogenesis of exosomes, their biomarkers are conventionally deemed to be ESCRT-associated proteins (Alix, TSG101, Syntenin, and HSC70), tetraspanin family proteins (CD9, CD63, and CD81), and major histocompatibility complex (MHC) class I and class II proteins. However, some of these proteins have also been demonstrated to be contained in other subtypes, such as flotillin-1, HSC70, and MHC class I and II proteins (40). Microvesicles derived from the plasma membrane mainly contain proteins present in the cytoplasm and plasma membrane, especially post-translational modified proteins (41). Apoptotic bodies contain high levels of apoptosis-associated proteins, such as cleaved caspase-3, C1q, and nuclear debris (42). Nevertheless, overlapping biomarkers exist among each group of EVs, making it imprecise for identification. In addition, isolation methods used in current studies, especially for microvesicles and apoptotic bodies, are mainly centrifugation based on the size and density of EVs, which leads to groups overlapping on the very edge of the size scale.

Exosomes and microvesicles transfer information and therapeutic molecules from viable cells to recipient cells (43), rendering them potential options for investigation in regenerative medicine. However, from the orthodox perspective, apoptotic bodies tend to be cell debris responsible for the clearance of dying cells (44). Moreover, cargoes distributed into apoptotic bodies vary in quantity and component (45, 46), along with a relatively large size scale, impeding the identification and mechanical exploration of apoptotic bodies. Hence, studies have paid more attention to the mechanisms and applications of the former two subtypes of EVs. In this review, our discussion of ADSC-EVs is mainly based on exosomes and microvesicles derived from ADSCs.

### Isolation and Characterization of EVs

**Figure 3:**
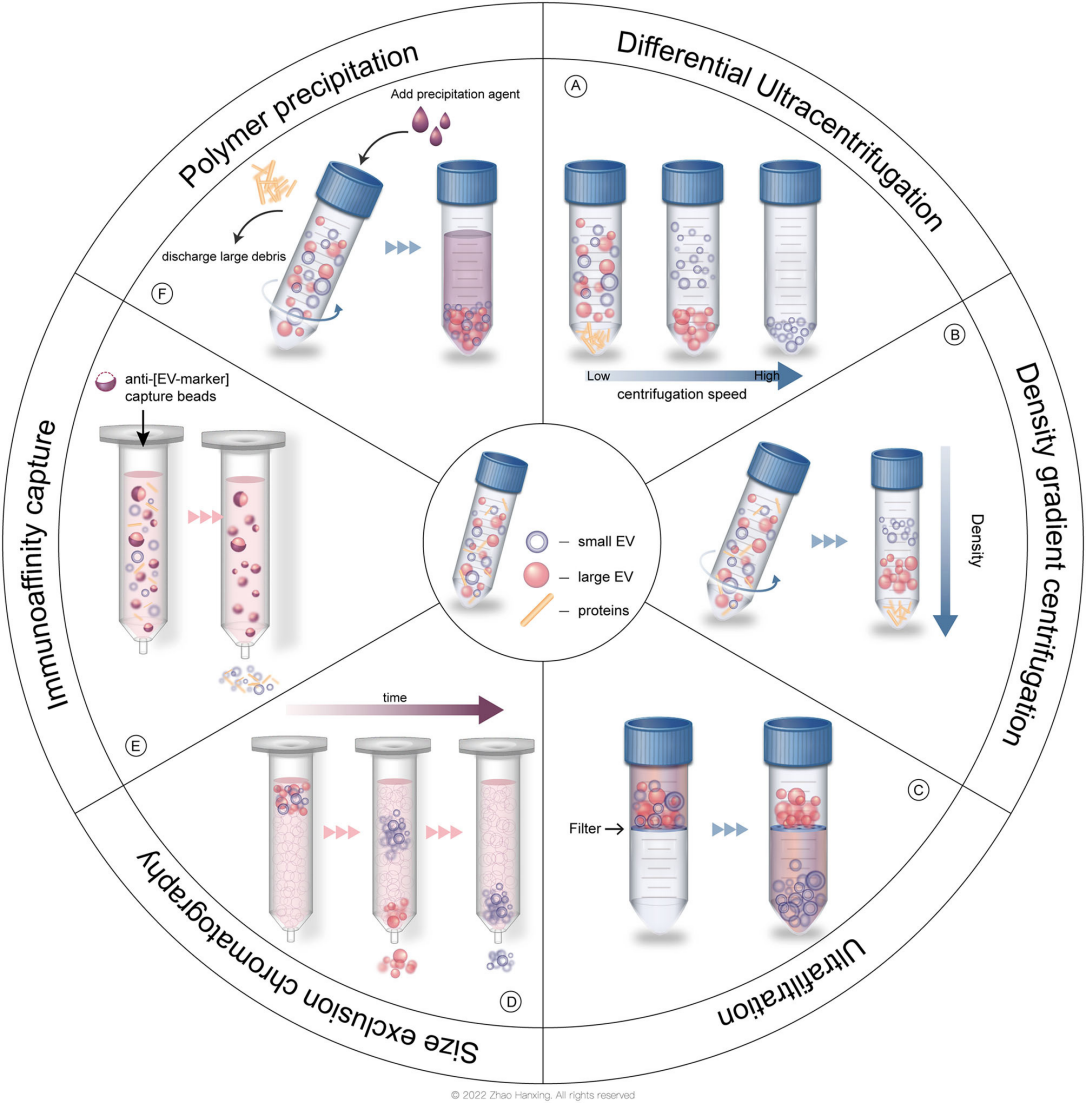
Common isolation strategies of EVs. **(A)** Differential ultracentrifugation (DC). **(B)** Density gradient centrifugation (DGC). **(C)** Ultrafiltration (UF). **(D)** Size exclusion chromatography (SEC). **(E)** Immunoaffinity capture (IC). **(F)** Polymer precipitation (PP).

In the past few decades, researchers have isolated EVs by several common strategies (Figure 3). The traditional method is centrifugation, which is based on the density of different groups of EVs, allowing denser particles to sediment out first. Differential ultracentrifugation (DC) is the most frequently used method and is still the “gold standard” for the isolation of EVs (26). Density gradient centrifugation (DGC) is an improved ultracentrifugation method that produces EVs with higher purity, in which a prepared density gradient generally formed by sucrose or iodixanol is required. EVs pass through a gradient with increasing density from top to bottom in the DGC system, and then each subtype of EVs is separated into perspective layers with different densities. Common methods based on the size of EVs include ultrafiltration (UF) and size exclusion chromatography (SEC) (28). In UF, the target group of EVs passes through the filtration membrane with a certain molecular weight cut off (MWCO) while larger particles are retained. In SEC, EVs with different sizes pass through the column filled with porous polymer microspheres that allows smaller particles to penetrate. Routes in those pores take more time for smaller EVs to elute than larger EVs. Immunoaffinity capture (IC) technology relies on the binding between antigens on the surface of EVs and antibodies attached to the surface of tools, such as magnetic beads or plates. IC allows the isolation of EVs originating from a specific source with certain surface proteins. Polymer precipitation (PP), typically polyethylene glycol base, takes advantage of strong hydrophilicity to “grab” the water molecules in the solution, rendering EVs “dehydrated” to aggregate (41). Commercial isolation kits with various strategies described above have also been used in research. However, each method possesses its shortcomings. A large initial volume is required for DC and a long DC duration increases the risk of structural damage to EVs and protein contamination. DGC is associated with extra preparation and a low yield of EVs. When EVs pass through the filter membrane, the pores might be blocked, causing low yield, and the shear force leads to deformation and lysis of EVs. SEC and IC cannot process a large volume of solution. EVs isolated by PP tend to be contaminated by polymers and proteins (41). The isolation method, which is linked to the purity of EVs, is suggested to be chosen according to different research purposes (26).

With the boosted development of technology, novel strategies have been developed to isolate EVs efficiently. Microfluidic techniques allow the isolation of EVs considering their physical and biochemical properties simultaneously. Acoustic (47), electrical (48), and electromagnetic field forces (49) can be addressed inside microfluidic devices to isolate EVs, along with immuno-based (50) and asymmetric flow field flow-based (51) microfluidic techniques. While this technology is still developing, the efficiency, simplicity, and low initial volume of the sample render it a promising method for future clinical application. Other methods of large-scale EV production include the use of bioreactor culture of parental cells, in which the characteristics of EVs need to be clarified with the culture condition in the bioreactor (52). Shear stress and cell extrusion can scale up EV-like vesicles production, while the purity of vesicles is relatively poor (53). Hydrostatic filtration dialysis and cytochalasin B induced vesicles have also been reported to improve EVs yield (54, 55). Immortalization of MSCs is also a potential strategy, along with increased safety concerns (56).

For the characterization of EVs, each subtype has been identified by some detection strategies based on their morphology, size, biomarkers of surface, and contents. The morphology of EVs is frequently observed by scanning electron microscopy and transmission electron microscopy (26). Electron cryo-microscopy and atomic force microscopy are also used (28). The size distribution and concentration of EVs are usually detected by nanoparticle tracking analysis and dynamic light scattering, and tunable resistive pulse sensing (57). Some new technologies, such as light microscopic single EV analysis, have been utilized to analyze the properties of a single EV (58). Common biochemical analysis methods of EVs include western blotting, flow cytometry, and liquid chromatography and mass spectrometry. In recent years, new technologies for EVs analysis have emerged, including small particle flow cytometry, micronuclear magnetic resonance, and thermophoretic profiling (33).

## Mechanisms by Which ADSC-EVs Play a Therapeutic Role in Skin Regeneration

**Figure 4:**
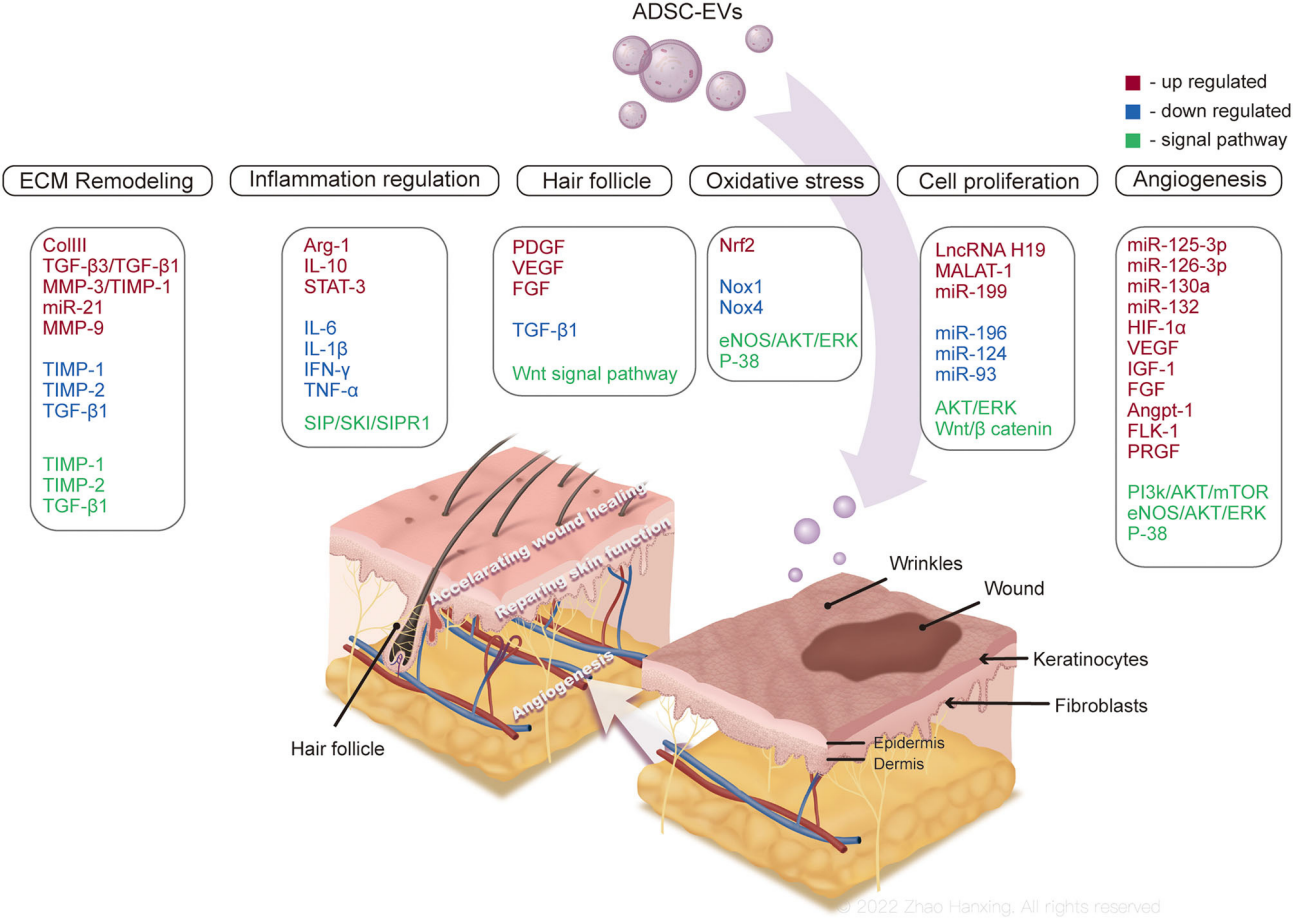
Mechanisms of ADSC-EVs promoting skin regeneration. ADSC-EVs might accelerate skin wound repair by participate in inflammation, angiogenesis, cell proliferation, and extracellular matrix (ECM) remodeling, regulating cell apoptosis and autophagy, and relieving oxidative stress in wound microenvironment. The regeneration of skin appendages and physiological functions are also promoted by ADSC-EVs.

Recent studies have demonstrated the beneficial role of ADSC-EVs in multiple tissue regeneration, such as skin (59), tendon (60), bone (61), and nerve tissue (62). ADSC-EVs exert a regenerative effect by delivering signals to cells with single or coordinated actions of biomolecules. Wound healing is one of the dominant components of skin regeneration and consists of several intricate and dynamic processes: hemostasis, inflammation, proliferation, and remodeling (63). ADSC-EVs participate in each process of wound healing, along with the regeneration of skin physiological functions to achieve ideal skin regeneration. The comprehensive abstract of current understandings of ADSC-EVs functioning in skin regeneration is shown in Figure 4.

### Promote Wound Healing

#### Inflammation Regulation

In the process of ideal wound healing, the immune system is supposed to defend against the invasion of pathogens by a moderate inflammatory response. When immune homeostasis is compromised, excess and persistent inflammation contributes to impaired wound healing, such as in chronic diabetic wounds (64). ADSC-EVs improve the inflammatory microenvironment at wound sites by regulating the activities of immune cells, thereby accelerating wound repair.

ADSC-EVs can regulate the balance between CD4 T cell subsets (65) and inhibit the proliferation of T cells and the release of inflammatory factor IFN-γ (66). The engulfment of ADSC-EVs by macrophages and subsequent increased expression of Arg-1 and IL-10, soluble markers of anti-inflammatory phenotype macrophages (M2) (67), were observed in obese mice. Mechanically, ADSC-EVs induced transactivation of Arg-1 in macrophages by transferring signal transducer and activator of transcription 3 (68). Additionally, the polarization of M2 macrophages after treatment with ADSC-EVs was found to be associated with the S1P/SK1/S1PR1 signaling pathway, along with reduced expression of inflammatory cytokines IL-6, IL-1β, IFN-γ, and TNF-α (69).

The immunosuppressive activity of ADSC-EVs regulates inflammation in skin wounds, too. Interestingly, another study demonstrated that ADSC-EVs attenuated the polarization of inflammatory M1 macrophages, while hardly inducing M2 polarization (70). Although clarification of this controversy needs to be addressed in further investigation, both inhibited M1 polarization and increased M2 polarization led to improved inflammation. In addition, treatment of ADSC-EVs in ischemia-reperfusion skin flaps resulted in less infiltration of inflammatory cells and ameliorated apoptosis (71).

Current studies have demonstrated the effects of ADSC-EVs as mediators in the inflammatory response, in which the inhibition of inflammation dominates, promoting wound repair. However, it is noteworthy that a necessary inflammatory response also contributes to the protection of the wound microenvironment (72). In the early stage of using ADSC-EVs, they were indicated to cause inflammation, whereas exert pro-adipogenic function and promote collagen synthesis in the late stage (73). From this perspective, homeostasis of inflammation at wound sites needs to be considered in both research and application of ADSC-EVs, rather than absolute inhibition of inflammation.

#### Angiogenesis

Angiogenesis involves multiple cytokines and intricate signaling pathways, which are essential for the supply of oxygen and nutrients as skin wounds heal. ADSC-EVs have been documented to trigger angiogenesis by transferring contained proangiogenic mediators. Neovascularization of human umbilical vein endothelial cells (HUVECs) was promoted with the treatment of ADSC-EVs, which was more robust when miR-126-3p was overexpressed (74). Other microRNAs carried by ADSC-EVs were also reported to participate in angiogenesis, such as miR-126, miR-130a, and miR-132 (75). Additionally, miR-125a-3p from ADSC-EVs enhanced angiogenesis of HUVECs by activating the PI3K/AKT signaling pathway, targeting the PTEN gene (76). Another study indicated that ADSC-EVs also contributed to angiogenesis in the diabetes microenvironment by increasing HIF-1α and VEGF expression through the PI3K/AKT/mTOR signaling pathway (77). The angiogenic potential of ADSC-EVs might be enhanced in the specific microenvironment. Under hypoxia conditions, ADSC-EVs encapsulated more proangiogenic growth factors, including IGF-1, FGF, VEGF and their receptors (17), angiopoietin-1, and fetal liver kinase-1 (78), and demonstrated more prominent neovascularization and faster wound repair. With the stimulus of PDGF, secretion of ADSC-EVs increased, along with upregulated c-kit and SCF (79). The c-kit is a tyrosine kinase receptor that regulates the differentiation of progenitor cells to blood or vascular endothelial cells. C-kit ligand SCF is a kind of stem cell regulator that plays an important role in angiogenesis and recruitment of MSCs (80). EVs released by ADSCs overexpressing glyoxalase-1 (GLO-1) promoted capillary growth compared with normal ADEC-EVs under high-glucose conditions by upregulating the eNOS/AKT/ERK/P-38 signaling pathway, which regulates the proliferation and migration of HUVECs (17).

ADSC-EVs facilitate angiogenesis by providing cargos that participate in capillary growth or activate angiogenic signaling pathways, thereby promoting wound healing. However, newly formed capillaries are supposed to degrade to form an appropriate vascular density similar to normal skin at the late stage of wound healing without scarring (81). In wound repair, further experiments are necessary to figure out optimal administration time and quantity of ADSC-EVs, avoiding potential side effects, such as scar formation out of excessive angiogenesis caused by the overdose of ADSC-EVs.

#### Cell Proliferation

During the proliferative phase of wound healing, epithelialization occurs mainly by proliferating and migrating to the wound site of epithelial cells. Fibroblasts are activated to proliferate and produce ECM to repair the defect. ADSC-EVs can be engulfed by human skin fibroblasts (HSFs) and HaCaT keratinocytes, promoting subsequent proliferation and migration (74, 82) in a dose-dependent manner (83). After the uptake of ADSC-EVs, the cell cycle of HSF was stimulated to accelerate re-epithelialization (82, 83) by the activation of AKT and ERK signaling pathways (82). Intriguingly but predictably, the Wnt/β-catenin signaling pathway, which participates closely in cell growth and renewal, was also involved in the proliferative effect of ADSC-EVs (84, 85). Long non-coding RNA (lncRNA) H19 in ADSC-EVs combined with miR-19b and inhibited its expression, targeting SRY-related high-mobility-group box 9, thus promoting wound healing (84). ADSC-EVs promote the proliferation and migration of human dermal fibroblasts (HDFs) and HaCaT keratinocytes by lncRNA MALAT-1 targeting miR-124 (85). Additionally, boosted proliferation and migration of HDFs were induced by upregulated miR-199 and downregulated miR-93 contained in ADSC-EVs (86). Finally, ADSC-EVs-treated M2 macrophages contributed to the proliferation and self-renewal of ADSCs (68).

#### Extracellular Matrix Remodeling

The synthesis and remodeling of ECM affect the formation of hypertrophic scars and the time of wound healing. Scar, which is composed of ECM, mostly collagen I (87), provides temporary strength to injured skin and will be degraded by matrix metalloproteinases (MMPs) gradually in the wound healing process (88). ADSC-EVs have been demonstrated to regulate the process of ECM remodeling. With the treatment of ADSC-EVs, deposition of collagen I and III with a well-organized histological structure increased at the wound site, along with the regeneration of skin appendages (89, 90). ADSC-EVs facilitated ECM remodeling during wound repair by upregulating the ratio of collagen III/I, TGF-β3/TGF-β1, and MMP-3/tissue inhibitors of MMP-1 (TIMP-1). The differentiation of fibroblasts to myofibroblasts that contribute to scarring was also inhibited by ADSC-EVs (91). Overexpressed miR-21 in ADSC-EVs promoted the expression of MMP-9 and MMP-3 and suppressed that of TIMP-1, TIMP-2 and TGF-β1 by activating the PI3K/AKT signaling pathway to restrain scar formation (92). Although persistent high MMPs levels indicated extra ECM degradation and poor prognosis in diabetic wounds (93), ADSC-EVs boosted the deposition of collagen, which is essential for ECM formation in skin wounds, exerting a positive effect on ECM remodeling (94). In the mouse model, ADSC-EVs were reported to increase the synthesis of collagen to accelerate wound repair, which was inhibited in the late stage to reduce scarring (83). However, with the treatment of ADSC-EVs, HSF produced less collagen I, collagen III, and α-SMA, which differed from other studies, although they all ameliorated scar formation (95). This controversial result might be because that HSF in this study was isolated from hypertrophic scar tissue, in which HSF remained persistently hyperactive (96) while other studies utilized cells from normal skin tissue.

#### Autophagy and Apoptosis Modulation

From the orthodox perspective, autophagy and apoptosis were merely considered to be essential parts during skin wound healing. However, some studies have documented that ADSC-EVs promote wound repair with autophagy and apoptosis involved in the microenvironment of the wound site. Overexpressed circular RNA mmu_circ_0000250 in ADSC-EVs suppressed the expression of miR-128-3p in endothelial progenitor cells (EPCs), hence activating autophagy of EPCs and attenuating apoptosis of skin tissue (16). Moderate autophagy has been demonstrated to augment angiogenesis by recovering the function of EPCs (97), thus promoting wound healing. ADSC-EVs also ameliorated apoptosis of skin cells under irritations, acting as a protective buffer in the wound microenvironment (17, 98). Interestingly, MSCs underwent remarkable apoptosis after transplantation *in vivo*, but they still exerted prominent therapeutic effects and prevented hypertrophic scar formation (99). ADSCs might participate in wound healing by secreting EVs in healthy conditions, but also transferring therapeutic information when they are dying. ADSC-EVs produced during apoptosis are potential functional parts, which need further investigations.

#### Oxidative Stress Relief

The generation of reactive oxygen species (ROS) in the processes of wound healing is required to defend against the invasion of pathogenic microbes and active cell survival signaling (100). Nevertheless, excessive ROS in the microenvironment of skin wounds causes oxidative damage and impaired wound healing. For example, in diabetic wounds, persistent high glucose level activates protein kinase C in smooth muscle and endothelial cells, increasing the activity of NDPH and the production of ROS, which leads to impairment of the viability of dermal fibroblasts and keratinocytes (64). After long-term exposure to high glucose, endothelial cells tend to reduce the secretion of vasoactive factor endothelial nitric oxide synthase (eNOS), resulting in restricted blood flow and difficult wound healing (101). ADSC-EVs have been investigated to exert protective effects in such an oxidative stress microenvironment, maintaining the biological activities of cells. ADSC-EVs relieved ROS damage in EPCs induced by high glucose via the reduced expression of oxidative stress-related proteins NOX1 and NOX4, which could be inhibited by the EV inhibitor GW4869 and enhanced by overexpression of nuclear factor erythroid 2-related factor 2 (102). When facing oxidative stress caused by hydrogen peroxide (H_2_O_2_), ADSC-EVs also maintained the viability and metabolic activity of keratinocytes and HSF (18), which might be attributed to lncRNA MALAT-1 contained in ADSC-EVs (85, 103). Additionally, EVs derived from ADSCs pretreated with H_2_O_2_ possessed a more prominent effect on oxidative stress relief and microvessel formation (71). Some studies have documented that pretreatment with H_2_O_2_ enables MSCs to produce higher levels of miR-21 to relieve cell death caused by oxidative stress (104, 105). Irritation seems to change the cargos of ADSC-EVs, thereby affecting their functions. GLO-1 is the rate-limiting enzyme in the glyoxalase system that plays an important role in the detoxification of advanced glycation end products (106, 107), accelerating the clearance of ROS in endothelial cells (108). After coculture with ADSC-EVs containing GLO-1, HUVECs accumulated less ROS and inflammatory cytokine IL-1β in a high glucose wound environment, with the involvement of the eNOS/AKT/ERK/P-38 signaling pathways (17).

### Regeneration of Skin Physiological Function

The closure of skin wounds is not the end of perfect skin regeneration, in which the recovery of normal structure and physiological function are also important. The barrier function of the skin mainly relies on the external layer, the epidermis that lies openings of appendages (hair follicles, sweat glands, and sebaceous glands) and intercellular lipids (ceramides, filaggrin, and cholesterol) (109–111). Recent studies have demonstrated that ADSC-EVs function as positive regulators in recovering skin physiological function, at least partly.

#### Hair Follicle Regeneration

Hair deficiency is one of the major aesthetic complaints not only in patients with alopecia but also in those who have healed skin wounds without hair follicle regeneration. Moreover, skin with more hair follicles heals faster than that with less hair or without hair, which is due to the involvement of hair follicle stem cells in wound healing (112, 113). However, wound repair of adult mammals is likely to form scars without skin appendage regeneration (114). ADSC-EVs seemed to rescue hair regeneration in some way. In nude mice models, additional 50 μg/ml ADSC-EVs in experimental groups were grafted with dermal cells and epidermal cells to skin wounds, in which more hairs with normal structure and mature hair follicles were observed, along with higher expression of PDGF and VEGF and lower TGF-β1 expression in skin tissue (20). Although morphological observation cannot provide a detailed explanation mechanically, PDGF and VEGF are deemed to be growth regulators of hair follicles in the anagen phase (115). In addition, the reduced expression of TGF-β1 might contribute to hair maintenance, since it participates in the catagen phase of hair development and affects hair follicle apoptosis-associated molecules (116, 117). ADSC-EVs themselves also contain cytokines that stimulate hair follicle growth, including VEGF and FGF (17, 118), and enable the activation of the Wnt signaling pathway essential to hair follicle induction (85, 98, 119, 120). Even so, more research in this field is required to decipher the specific molecular mechanism of hair follicle regeneration promoted by ADSC-EVs. Current studies are largely based on rodent models, however, the structure of the skin and mechanisms of wound healing in humans and rodents are different.

#### Skin Barrier Repair

The epidermal barrier of the skin is supplied by stratum corneum (SC), which consists of corneocytes and an intercellular lipid mixture of ceramides, free fatty acids, and cholesterol (111). Among lipids in SC, ceramides are the dominant content, with weight over 50% (121), the defect of which is a critical part of etiology in atopic dermatitis (AD). Recently, ADSC-EVs have been indicated to promote skin barrier repair in AD mouse models. With the injection of ADSC-EVs, impaired SC hydration induced by oxazolone was normalized. Meanwhile, the quantity of long-chain dihydroceramide, a precursor component during de novo synthesis of ceramides, was significantly increased (19, 122). Predictably, enhanced synthesis of sphingosine-1-phosphate (S1P), a metabolite of ceramides, was also observed in this study. S1P has been reported to inhibit ceramide-associated apoptosis and stimulate the viability and differentiation of keratinocytes (123, 124). As mentioned before, ADSC-EVs activated the S1P / SK1 / S1PR1 signaling pathway to promote M2 macrophages polarization, thus attenuating inflammation (69), which is in accordance with the reduced inflammatory cytokines, such as TNF-α and IFN-γ (19). The lipid regenerative function of ADSC-EVs might be partly due to their regulatory role in inflammation. The particular actions of contents in ADSC-EVs helping skin barrier repair remain elusive, but ADSC-EVs may be a potential cell-free therapeutic approach for regeneration of skin barrier function.

## Preclinical and Clinical Studies of ADSC-EVs for Skin Regeneration and Repair After Injury

### Wound Healing

After long-term exploration, researchers gradually found the advantages and appropriate drug delivery methods of ADSC-EVs in skin regeneration and repair. Initially, the application of human fibrocyte-derived EVs in wound models of diabetic mice showed that all wound healing was significantly enhanced (125). EVs derived from other MSCs were also found to play a critical role in promoting re-epithelialization, collagen synthesis and angiogenesis in skin wound healing (126, 127). Considering that it would be easier to obtain autologous ADSC when it is finally applied to human wound treatment because liposuction has been very common and mature, researchers mixed ADSC-EVs with fibroblasts and demonstrated that this synergistic effect was helpful to induce the enrichment of miRNAs related to promoting wound healing in fibroblasts (86). Earlier it was shown that direct IV administration of ADSC-EVs in a murine wound model was beneficial to ameliorate cutaneous repair by regulating ECM remodeling (91). Furthermore, local injection of ADSC-EVs into mouse full-thickness cutaneous wounds significantly increased re-epithelialization, collagen deposition, and neovascularization and induced accelerated wound closure (82). To explore the therapeutic potential of ADSC-EVs more accurately, BMSC-EVs and ADSC-EVs were applied, respectively to diabetic wounds, and the results demonstrated that ADSC-EVs possess the more potent pro-angiogenic activity and can promote the wound healing of diabetic ulcers (13). Considering the availability of adipose tissue and fewer ethical concerns of EVs, emerging skin regenerative studies tend to focus on ADSC-EVs to better transform to future clinical trials (128).

Since the regeneration and repair mechanism of swine skin is similar to that of humans, swine skin is more promising than rodent skin for studying skin wounds. The topical conditioned medium of ADSC therapy displayed increased angiogenesis and a diminished inflammatory response and improved the wound closure rates in the full-thickness dorsal wound models in pigs (129). However, such research reports are very scarce. We analyze the possible reasons from two aspects. On the one side, it is technically and economically more difficult to operate diabetic or burn wound models in large animals. On the other side, the application of EVs in large animals requires a larger dose of EVs, which is limited by current EV isolation methods. Researchers need to accelerate the maturation of methods producing large-scale EVs or find new delivery methods to improve local retention of EVs, which is more conducive to the research on EVs application in the future.

At the time of this review writing, only a few trials related to applying EVs to treat skin wounds can be searched on the web clinicaltrials.gov (accessed on Nov. 20, 2021). Unfortunately, no trial related to ADSC-EVs is included until now, however, the following summary of other EVs applied to skin wound repair has important reminder and reference values for the subsequent direct application of ADSC-EVs in this field. Autologous serum-derived EVs will be evaluated to determine whether they could play a positive role in cutaneous wound healing (NCT02565264) and venous ulcers not responsive to conventional treatments (NCT04652531). One trial will investigate the therapeutic potential of stem cell-conditioned medium as an additional growth factor in chronic skin ulcer healing (NCT04134676). Another trial that has completed patient recruitment aims to develop a safe and reasonable method of administering BMSC-EVs to burn wounds (NCT05078385). Although no EV product had been approved by the FDA to date, it is firmly believed that more clinical studies will be included soon, and high-quality clinical trial results can energetically promote the final clinical application of EVs.

### Skin Photoaging and Senescence

As the most commonly used stem cell therapeutics, the application of ADSCs in skin rejuvenation has been widely investigated. The paracrine effects of ADSCs, which are characterized by the release of cytokines in the form of EVs, are recognized as critical mechanisms in skin tissue repair and regeneration. Drawing support from their paracrine effects, ADSC-free derivatives, including ADSC-EVs and ADSC conditioned medium (ADSC-CM), have gained attention as novel therapeutics in ameliorating skin health (24, 130). As is universally acknowledged, human dermal fibroblasts (HDFs) work as essential objects in anti-wrinkle, wound healing, skin aging, and overall homeostasis studies. The ADSC-EVs and ADSC-CM acted as therapeutic agents in skin rejuvenation by improving proliferation, migration, and collagen synthesis in HDFs without adverse effects (131, 132). Because ADSC-CM could effectively downregulate the activation and transcription of UVB-related signaling pathways and upregulate antioxidant response agent expression, it was regarded to play a positive role in keeping HDFs away from UVB-induced photoaging damage (133). By decreasing ROS production and MMPs overexpression, which are directly linked to ECM proteins degradation, ADSC-EVs slowed wrinkle formation and skin photoaging (70, 134). Morphometric and morphological assessment of histological changes showed that ADSC-EVs could decrease UV-mediated epidermal thickening and prevent skin damage caused by UV photoaging (135). Because research on ADSC-EVs is still limited, the majority of studies on the therapeutic function of ADSC derivatives in skin rejuvenation focus on ADSC-CM. Despite slight differences in protein properties that exist between ADSC-EVs and ADSC-CM, these two main components in ADSC derivatives contain factors linked to ECM remodeling and immunoregulation, which are crucial for maintaining skin homeostasis and antiaging (136). Furthermore, the removal of EVs from ADSC-CM significantly weakened its positive effects on cell proliferation, migration, and scar prevention (137, 138). This demonstrates that EVs are crucial components in ADSC-CM and may possess an independent or synergistic role that is beneficial for anti-skin photoaging.

### Skin Pigmentation After Trauma

In posttraumatic skin regeneration, the non-Caucasian race is more susceptible to pigmentation and/or scar formation. For such patients, anti-scar treatment while desalinating the pigment as far as possible will be conducive to the appearance of regenerated skin closer to normal. The increased expression of S1P induced by ADSC-EVs was negatively correlated with the production of melanin, which implies that ADSC-EVs have potential application value in skin-brightening (19). As shown in a prospective, double-blind, randomized, placebo-controlled study, a cosmetic formulation containing ADSC-EVs decreased skin melanin contents and reversed hyperpigmentation in human volunteers (139). Although the effect of ADSC-EVs on improving skin brightness becomes weak with time due to the limitation of transdermal delivery, the actuation duration of ADSC-EVs will be expected to be ameliorated with the continuous development of new drug delivery agents such as nano biomaterials. In the early stage of skin injury repair, the application of ADSC-EVs can promote scarless healing (83), but whether similar effects will appear in colored people and whether they could dilute pigmentation while reducing scarring need to be further studied.

Due to the different types of skin damage, the diversity of the involved skin layers also exists. Further research will be needed to locate which cells in the skin damage microenvironment are targets of ADSC-EVs. It is foreseeable that accurately applying EVs to selectively act on target cells in the epidermis or dermis will greatly contribute to further explaining the specific mechanism of EVs' regenerative function.

## Challenges and Prospects

ADSCs have been demonstrated to play a beneficial role in skin regeneration and rejuvenation due to their participation in multiple biological activities of skin cells. ADSC-EVs, the products, and the information disseminators of ADSCs, seem to inherit similar therapeutic effects from their parental cells but are safer and more convenient to use. ADSC-EVs may promote skin regeneration, including structural repair and functional recovery, by accelerating the canonical wound repair process and regulating the skin microenvironment. The regenerative effect of ADSC-EVs renders them a potential option for clinical application in wound treatment and skin cosmetology. With multiple signal recognition molecules anchoring to the natural lipid membrane, ADSC-EVs are underlying carriers delivering drugs *in vivo*. EVs have been modified to carry therapeutic molecules by multiple loading methods, such as transfection of parental cells for endogenous loading (16), electroporation (140), co-incubation (141), and freeze-thawing (142) for exogenous loading. Furthermore, the combination between ADSC-EVs and bioactive scaffolds is an effective strategy to improve the quick clearance of ADSC-EVs at the wound site. Meanwhile, hydrogel dressings containing ADSC-EVs can be modified to possess functions promoting wound repair, such as antibacterial (89) and antioxidation (18).

Nevertheless, as mentioned above, current comprehension of ADSC-EVs themselves and the mechanisms of their actions remain elusive, and challenges exist in their manufacture and application. With skin structure more similar to human skin, pigs and guinea pigs are preferred animal models for skin wound research than rodent models mostly used in current studies of ADSC-EVs in skin regeneration. More convincing evidence needs to be uncovered to clarify the current enigma existing in the mechanisms of ADSC-EVs. For example, regulation of ADSC-EVs on the proliferation and differentiation of fibroblasts, which is essential for wound repair but results in scar formation when overwhelming. From a current perspective, the effect of ADSC-EVs is strongly associated with their contents, which are influenced by the physiological conditions of their parental cells (14, 143). Diverse isolation methods also lead to discrepancies in cargo (25). Quality control of ADSC-EVs is fundamental to probe the particular portion of ADSC-EVs functioning directly or mediately as key therapeutics in skin regeneration, thus further formulating instructive protocols to study or manufacture in the future. Likewise, a low yield of ADSC-EVs is also a crucial issue, which is attributed to the limited culture medium of ADSCs and repeated centrifugation processes in conventional isolation methods. Strategies for the production with large quantities and long-term storage of ADSC-EVs must be developed for clinical application. To date, research on ADSC-EVs has mainly remained at the laboratory level. To utilize the regenerative and therapeutic functions of ADSC-EVs, much more comprehensive information needs to be uncovered.

## Author Contributions

YW, LC, and ZZ: conceptualization. YW and ZZ: validation. YW, LC, HZ, ZL, and JC: investigation. YC and ZZ: resources. YW: original draft preparation. LC and ZZ: review and editing. HZ, YC, and JC: visualization. ZZ: supervision and project administration. ZZ and ZL: funding acquisition. All authors have read and agreed to the published version of the manuscript, accepted responsibility for the entire content of this manuscript, and approved its submission.

## Funding

This work was supported by grants from the National Natural Science Foundation of China (No. 81871574), and Scientific Research Projects of Sichuan Health Commission (No. 19PJ097).

## Conflict of Interest

The authors declare that the research was conducted in the absence of any commercial or financial relationships that could be construed as a potential conflict of interest.

## Publisher's Note

All claims expressed in this article are solely those of the authors and do not necessarily represent those of their affiliated organizations, or those of the publisher, the editors and the reviewers. Any product that may be evaluated in this article, or claim that may be made by its manufacturer, is not guaranteed or endorsed by the publisher.
